# Salivary Cortisol, but Not Oxytocin, Varies With Social Challenges in Domestic Pigs: Implications for Measuring Emotions

**DOI:** 10.3389/fnbeh.2022.899397

**Published:** 2022-05-23

**Authors:** Liza R. Moscovice, Ulrike Gimsa, Winfried Otten, Anja Eggert

**Affiliations:** ^1^Psychophysiology Unit, Institute of Behavioural Physiology, Research Institute for Farm Animal Biology (FBN), Dummerstorf, Germany; ^2^Service Group Statistical Consulting, Institute of Genetics and Biometry, Research Institute for Farm Animal Biology (FBN), Dummerstorf, Germany

**Keywords:** physiology, non-invasive, emotions, *Sus scrofa domesticus*, weaning, social isolation, reunions, play

## Abstract

Animals respond to inherently rewarding or punishing stimuli with changes in core affective states, which can be investigated with the aid of appropriate biomarkers. In this study we evaluate salivary cortisol (sCORT) and salivary oxytocin (sOXT) concentrations under baseline conditions and in response to two negatively- and two positively-valenced social challenges in 75 young pigs (*Sus scrofa domesticus*), housed and tested in eight social groups. We predicted that: (1) Relative to baseline, weaning and brief social isolation would be associated with increases in sCORT, due to psychosocial stress, and reductions in sOXT, due to a lack of opportunities for social support; and (2) Opportunities for social play, and reunions with group members after a separation would be associated with weaker sCORT responses, and increases in sOXT concentrations compared to baseline and to negative social challenges. Testing and sample collection occurred between 28 and 65 days of age and involved a within-subject design, in which every subject was sampled multiple times in neutral (baseline), negative and positive social contexts. We also recorded behavioral data and measured rates of agonism, play and affiliative interactions in the different contexts, prior to saliva sampling. As expected, negative social challenges were associated with robust cortisol responses. Relative to baseline, pigs also had higher sCORT responses to positive social challenges, although these differences were only significant during reunions. Salivary oxytocin concentrations did not differ between the different social conditions, although sOXT was lowest during the brief social isolation. Behavioral analyses confirmed predictions about the expected changes in social interactions in different social contexts, with increases in agonism following weaning, increases in coordinated locomotor play in the play context and high rates of affiliative interactions during reunions. Relative sCORT reactivity to different contexts may reflect the intensity of emotional responses, with greater increases occurring in response to challenges that involve more psychosocial stress. Our results suggest that sOXT is not a reliable indicator of emotional valence in pigs, although more research is needed to characterize sOXT responses to various challenges with and without access to social support.

## Introduction

From an evolutionary perspective, emotions are viewed as coordinated short-term changes in behavior, physiology and cognition that help individuals to avoid harmful situations that may decrease fitness, and to seek out positive and rewarding experiences that enhance fitness (reviewed in: Mendl et al., 2010; Paul and Mendl, 2018). Threats to fitness can be caused by environmental risks, social aggression or social isolation, while benefits to fitness occur through access to important resources including food, safety and social partners. There is growing consensus that humans and other animals respond to inherently rewarding or punishing stimuli by exhibiting different core affective states (Burgdorf and Panksepp, 2006; Dolensek et al., 2020). These core affective states contain several measurable features (Anderson et al., 2014; Zych and Gogolla, 2021), including valence (emotional responses are negative or positive), scalability (emotional responses show gradations in intensity) and persistence (emotional responses outlast the stimuli), which can be investigated in animal models with the aid of appropriate biomarkers.

Among the physiological indicators that are frequently linked to animal emotions are the glucocorticoid (GC) hormones, which are released into circulation after activation of the hypothalamic-pituitary-adrenal (HPA) axis in response to physical or psychosocial stressors (reviewed in Sapolsky et al., 2000). The majority of research on the HPA axis has focused on GC responses to involuntary stressors that pose threats to fitness, such as restraint (rats: Pitman et al., 1988), forced exercise (rats: Wotjak et al., 1998; humans: Altemus et al., 1995); social aggression (rats: Neumann et al., 2001a) or social isolation (pigs: Ruis et al., 2001). As a result, increases in GCs in animals are often interpreted as indicators of distress in response to threats. However, the HPA axis is also involved in mobilizing energy to respond adaptively to various voluntary and fitness-enhancing challenges (reviewed in Buwalda et al., 2012). For example, exposure to environmental enrichment for captive animals (e.g. pigs: de Jong et al., 2000; rats: Konkle et al., 2010), voluntary exercise (humans: Hew-Butler et al., 2008), and sexual arousal (rats: Buwalda et al., 2012), are also associated with elevated GCs. The combined evidence cautions against using measures of GCs alone as indicators of emotional valence in animals. However, there is some evidence that relative changes in GCs in response to different challenges may reflect differences in perceived intensity of challenges for individuals (García et al., 2000; Hew-Butler et al., 2008; Koolhaas et al., 2011), suggesting that GCs may be useful for measuring the scalability of emotional responses.

The potential role of the neuropeptide hormone oxytocin as a bioindicator of emotional valence is increasingly discussed (Mitsui et al., 2011; Rault et al., 2017). From the hypothalamus, centrally-released oxytocin binds to regions of the social brain including the nucleus accumbens, where it enhances the rewarding aspects of social behavior (Landgraf et al., 2009) and the amygdala, where it reduces fear and anxiety in order to facilitate social interactions (Knobloch et al., 2012). Oxytocin also binds to receptors in the hypothalamus, where it can exert inhibitory effects on the HPA axis (rodents: Neumann et al., 2001b; primates: Parker et al., 2005). This evidence has led to the hypothesis that oxytocin is responsible for the buffering effects of social support on stress reactivity, which have been documented in a range of species (Engelmann et al., 2004; Hennessy et al., 2009). Oxytocin can also be released peripherally via the posterior pituitary, either in a coordinated manner with central release (e.g. Nyuyki et al., 2011), or independently (e.g. Neumann, 2002), depending on the context and intensity of the stimulus (Valstad et al., 2017). A large body of research indicates that exogenously-induced or endogenously-occurring elevations in circulating oxytocin are associated with increased trust, generosity and cooperation in humans (Kosfeld et al., 2005; Campbell, 2010), and increases in affiliative interactions in a range of species (humans: Gangestad and Grebe, 2017; primates: Crockford et al., 2013; rodents: Burkett et al., 2016). However, peripheral oxytocin concentrations also increase in response to threats to social relationships (Neumann, 2002; Campbell, 2008; De Dreu, 2012), and to a range of other psychosocial (e.g. Neumann et al., 2001a; Engert et al., 2016; Alley et al., 2019) and physical stressors (e.g. Hashiguchi et al., 1997; Wotjak et al., 1998; de Jong et al., 2015). More research is needed on the various contexts and types of stressors that elicit oxytocin release, in order to understand the interplay between the HPA-axis and the oxytocinergic system, and to evaluate the potential for oxytocin to be used as a bioindicator of emotional valence (reviewed in Rault et al., 2017).

In this study, we evaluate salivary cortisol (sCORT) and salivary oxytocin (sOXT) concentrations under baseline conditions and in response to negatively- and positively-valenced social challenges that are associated with strong behavioral responses in pigs. Pigs are highly social animals with close similarities in organ physiology and brain development to humans (Swindle and Smith, 2008). They exhibit evidence for advanced cognitive abilities (Croney and Boysen, 2021), emotional contagion (reviewed in Baciadonna et al., 2018) and spontaneous prosocial behavior (Masilkova et al., 2021). As such, we expect pigs to show emotional responses to different social challenges. We also measure social behavior and activity levels during the different social challenges, as additional indicators that the challenges were associated with different emotional responses.

As negatively-valenced social challenges, we measured responses to: (i) weaning and mixing into new social groups and (ii) brief social isolation. Both of these contexts remove pigs from the safety and security of stable social groups. Weaning is associated with increases in aggression and salivary cortisol concentrations when pigs from different litters are mixed together into new social groups (Colson et al., 2012), as is typical in commercial pig production settings (reviewed in Düpjan et al., 2021). Prolonged social isolation triggers increases in cortisol concentrations and negative health indices in young pigs of both sexes (reviewed in Gimsa et al., 2018), and we expect that even brief social isolation will trigger robust stress responses in immature pigs, relative to baseline measurements when pigs are in stable social environments. We further hypothesize that weaning and social isolation will be associated with reductions in sOXT, related to a lack of opportunities to seek out and receive social support. Alternatively, given evidence that peripheral oxytocin concentrations can increase in response to psychosocial stressors (e.g. Neumann et al., 2001a; Engert et al., 2016), it is also possible that negatively-valenced social challenges will trigger increased sOXT relative to baseline.

We also exposed pigs to two positively-valenced social challenges involving: (i) opportunities for play and (ii) reunions with their social group after a brief separation. Play behavior is associated with positive health outcomes in pigs and other mammals (e.g. Boissy et al., 2007; Lawrence et al., 2018), and is a promising area for identifying physiological markers of positively-valenced emotions. Play behavior is most commonly observed in pre-pubertal pigs (Newberry et al., 1988) and is reliably triggered when young pigs are exposed to larger and more enriched-environments (Wood-Gush and Vestergaard, 1991; Donaldson et al., 2002). Pig play behavior often combines social and locomotor components, in which several pigs simultaneously exhibit exaggerated scampering, hopping and/or pivoting movements that serve no apparent function (e.g. Newberry et al., 1988; Reimert et al., 2013). As a second positively-valenced social challenge, we expect that reunions with members of one's social group after a brief separation will be associated with short-term increases in positive social interactions involving the reunited individual, including prolonged licking and nibbling, which may serve functions in pigs that are similar to grooming in other species (Gonyou, 2009; Camerlink and Turner, 2013).

We predict that positive social challenges will also be associated with increases in sCORT relative to baseline, due to increases in activity levels and the associated energetic demands put on individuals. However, if sCORT reflects the scalability of emotional responses, then we expect pigs to have reduced sCORT reactivity to positive social challenges in comparison with their responses to negative social challenges. We expect that positive social challenges will be associated with increases in sOXT, due to increases in social behaviors that are mediated by the oxytocinergic system, including behavioral synchronization (Feldman, 2012; Jiang and Platt, 2018) during play and affiliative tactile interactions (Dunbar, 2010) during reunions. By measuring sCORT, sOXT and behavior in pigs in response to differently-valenced social challenges, we will investigate the usefulness of combining these non-invasive physiological indicators to assess the valence and intensity of emotional responses.

## Materials and Methods

### Animal Husbandry and Testing Procedure

Subjects were German Landrace pigs born and housed at the Experimental Pig Facility at the Research Institute for Farm Animal Biology (FBN) in Dummerstorf, Germany. Farrowing is synchronized among several sows. After birth, piglets are housed with their mothers in 6 m^2^ farrowing compartments with plastic floors and a covered, heated piglet nest area (0.90 m^2^). Piglets have unrestricted access to water and are offered dry food (HAKRA-Immuno-G; Una Hakra, Hamburg, Germany) in addition to milk beginning from 14 days of age. Piglets receive tattoos at 1 day of age and ear tags with identification numbers at 27 days of age. Pigs are not subjected to tail docking, teeth clipping, castration or other invasive procedures. At 28 days of age, subjects were weaned and mixed into new social groups, by randomly selecting pigs from two to three different litters to make groups of 9–10 individuals. Groups were sex-balanced and contained equal numbers of littermates from each contributing litter. Pigs were tested in four temporally-separated cohorts, with each cohort consisting of two social groups, for a total of *n* = 78 subjects (39 females and 39 males). Power analyses indicated that this sample size would be sufficient to detect true effects with 80% probability (see Supplementary Materials). Cohorts were housed in identical, neighboring 2.8 x 2.8 m pens in the same room, with partially slatted flooring and a sleeping mat. Pigs in neighboring pens could exchange auditory and olfactory cues, but did not have direct visual contact. Indoor lighting in the room was controlled on a timer, with lights on from 7:00–15:00 h. Pigs also received natural light through a window. Food (Porcistart, Trede und von Pein GmbH, Damfleth, Germany) and water were provided *ad libitum*, and straw and an enrichment toy (Easyfix® Luna 68, Ballinasloe, Ireland) were provided daily. Room temperature was maintained between 20 and 22°C. Pigs were regularly given back numbers using livestock spray paint to facilitate individual identification. All research procedures were approved under the German Animal Welfare Act (German Animal Protection Law, §8 TierSchG) by the Committee for Animal Use and Care of the Agricultural Department of Mecklenburg-Western Pomerania, Germany (permit LALLF 7221.3-1-036/20).

**Figure 1:**
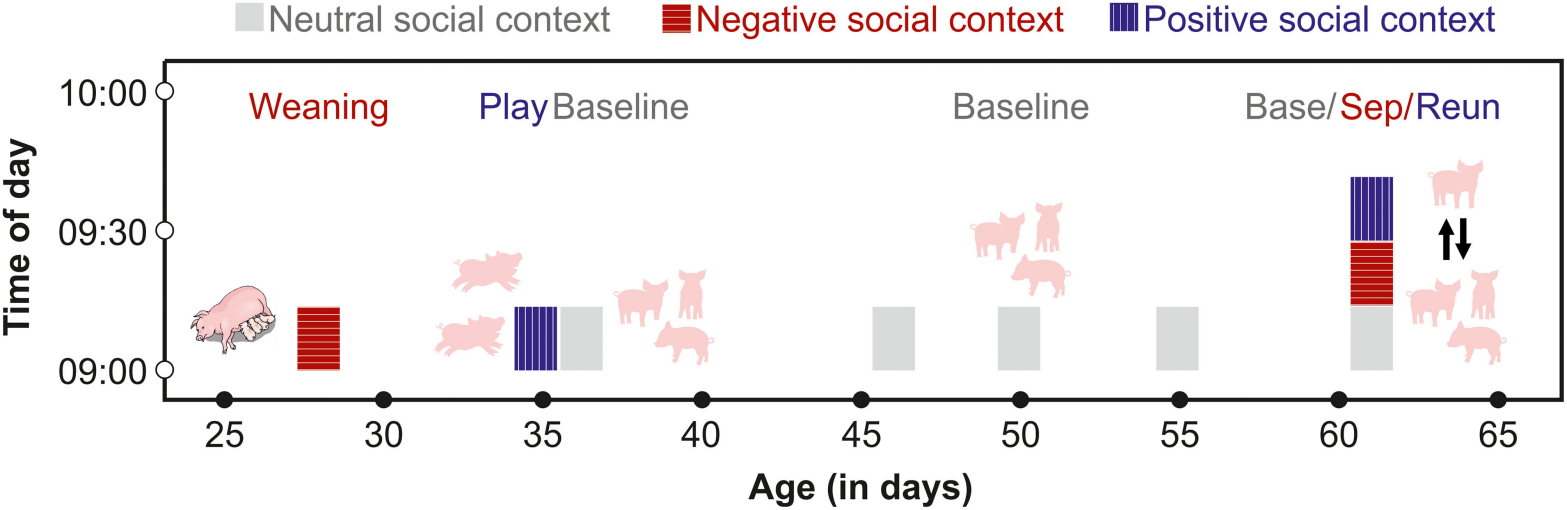
Schematic of the time frame for saliva sampling of each subject. From 28 to 55 days of age, pigs were sampled at similar times of day after exposure to negative, positive or neutral social contexts. From days 60 to 65, each pig was sampled three times on one day before (Base) and during a separation (Sep) from their group members, and after a reunion (Reun).

Testing and sample collection occurred from August 2020–February 2021, and involved a nested, within-subject design, in which each pig was repeatedly tested between 28 and 65 days of age in negative, positive and neutral (or baseline) social contexts. Of the 78 subjects, one died during the study period of unknown causes, and two subjects were removed from the study to be treated for illnesses. For these three subjects, we included only their initial baseline, weaning and play samples. The remaining *n* = 75 subjects experienced all of the positive and negative social conditions, and were also sampled multiple times under baseline conditions (Figure 1).

Cortisol has a strong diurnal rhythm, which typically peaks early in the morning, after first waking in pigs (Ruis et al., 1997). Little is known about the diurnal rhythm of oxytocin secretion in pigs, although oxytocin does not have a strong diurnal rhythm in other species (humans: Blagrove et al., 2012; Van Dam et al., 2018, goats: Seckl and Lightman, 1987). To avoid sampling during the waking cortisol response, we collected samples beginning at 8:30, 1.5 h after lights were switched on. We also sampled from each group at the same time of day following negative, positive and neutral social contexts. A few days prior to weaning, two assistants entered the farrowing compartments of future subjects and acclimated the piglets to their presence and to the process of chewing on swabs to provide saliva samples. At 28 days of age, pigs in a given cohort were weaned in a stratified manner, with the first group weaned at 7:30 and the second group weaned at 8:00. We collected saliva samples from each group one hour following weaning, at 8:30 and 9:00 respectively. The one hour interval before sampling was chosen to allow pigs to react to their new social environment, and to provide sufficient time for pigs to begin to exhibit post-mixing aggressive behaviors that are associated with the establishment of dominance hierarchies. After a week-long period of adjustment to their new social groups, the two groups were again tested in a stratified manner at 8:00 and 9:00 in a social play condition, during which each group was given free access to the large corridor (5 x 1.5 m) adjacent to their home pen. The corridor contained the same enrichment toy that was also available in the home pens. To initiate the play opportunity, a researcher entered the designated pen, propped one of the stall doors open and then left the room. Pigs then had free access to the corridor as well as to their home pens for 30 min. Two assistants then entered the room, returned the piglets to their home pen and collected saliva samples from the tested group, at 8:30 and 9:30 respectively. On the following day, baseline samples were collected from members of each group in their home pens, beginning at 8:30 or 9:00.

Between 46 and 55 days of age, we collected between 1 and 4 additional baseline samples from subjects. Baseline samples were collected between 8:30 and 12:00, and pigs were left undisturbed in their social groups prior to sampling. Following saliva sampling, pigs were exposed in their social groups to a cooperation task for a different project (Moscovice and Rault, unpublished data). Pigs did not receive any other interventions during this time period. Between 60 and 64 days of age, each subject underwent one 15-min separation from their social group in a pre-determined random order. Before the separation, an assistant entered the pen and collected a baseline sample from the subject. The assistant then opened the door and allowed the subject to leave the stall, which pigs did willingly without being handled. The assistant used a sorting board to encourage the pig to walk down a short hallway, until they entered an open door to a second experimental room, containing a 2.8 x 2.8 m arena. The arena contained an identical enrichment toy to the one available in the home stall, but was otherwise empty. The assistant closed the arena door and left the subject alone in the room for 15 min. The assistant then returned to the room, collected a post-separation saliva sample and led the pig down the hallway and back into their home stall, where they were left undisturbed for 15 min during the reunion phase. The assistant then entered the room and collected a post-reunion saliva sample from the subject. We tested two subjects per group per day and alternated between groups, so that each group had a recovery period of ~30–45 min between the end of a reunion and the next separation. By collecting multiple baseline samples over a month-long period that also involved different developmental stages and newer vs. more-established social groups, we hoped to gain a more robust measure of fluctuations in baseline hormone concentrations than would be possible by sampling at fewer time points (Martins et al., 2020).

### Behavioral Observations

Prior to collecting saliva samples, we recorded behavioral data from each group in each social context, using ceiling- and wall-mounted video cameras. One of the researchers (LRM) and two assistants coded behavioral data in Noldus® Observer XT 15 (Noldus Information Technology bv, The Netherlands). We recorded all occurrences of prolonged (> 3 s) agonism, affiliative interactions and locomotor play within groups in the 30 min before the weaning, play and first baseline hormone samples were collected. Agonism was defined by unilateral or reciprocal ramming, pressing and/or biting using the head and/or body, which was often accompanied by chasing by an aggressor, and avoidance behaviors by the eventual loser. For affiliative behavior, we focused on prolonged tactile interactions in which pigs used their nose or mouth to gently manipulate the skin or hair of partners. Locomotor play included primarily scampering (bouncy running), which was sometimes combined with pivoting (swiftly turning around to face in a different direction) and/or flopping on the ground. Unlike other forms of social play such as head butting and shoving, locomotor play is easy to disambiguate from agonism (Held and Špinka, 2011; Lawrence et al., 2018). We also measured the frequency of group locomotor play, which we defined when three or more individuals participated simultaneously in locomotor play. For each behavior, we measured the number of independent events within each group, defined as events that were separated by a > 10 s pause when they involved the same subjects. Behavior during weaning and play was analyzed in all eight groups, but behavior during baseline was analyzed in seven groups, due to technical issues with the video recording on the baseline day for one group. To determine general activity levels in different social contexts, we collected instantaneous scan samples of all visible pigs at two-minute intervals, with pigs who were standing or moving at the scan interval considered to be active. During the first 15 min of each reunion, we recorded focal continuous data on agonism and affiliative behavior between each reunited pig (*n* = 74) and other group members. Due to a technical problem with the video camera, we did not obtain behavioral data for one of the *n* = 75 reunion events.

### Hormone Collection and Analyses

Saliva samples were collected by one of the authors (LRM) and two assistants using SalivaBio® Infant Swabs (Salimetrics, CA, USA), which are designed to collect passive drool from human infants and have been validated for salivary hormone measurements in other species (e.g. dogs: MacLean et al., 2018). Each swab was secured to a wooden dowel, for ease of handling. All subjects were familiar with the sample collectors and had been previously habituated to sample collection. During collection, two researchers entered the pig stall, presented a swab to a subject chosen at random and encouraged them to mouth and chew on the swab for between 1 and 2 min. During this time the assistants prevented other pigs from attempting to manipulate the same swab. Pigs were not actively handled or restrained during sampling, and all samples were provided voluntarily. Saturated swabs were inspected and discarded if there were any signs of blood on the swabs. Swabs were placed in polypropylene tubes on ice immediately after collection, and were centrifuged within 30 min of collection at 4°C (15 min, 2,000 × g). The eluent was stored at −80°C until hormone analyses.

Due to individual variation in the amount of saliva produced, not all collected samples had sufficient volumes to analyze both hormones. For samples with > 300 μl (*n* = 437), both sCORT and sOXT were measured. For samples with 200–300 μl (*n* = 20), only sOXT was measured. For samples with <200 μl (*n* = 87) only sCORT was measured. Prior to sOXT measurement, we performed a solid phase extraction following a protocol modified from Lürzel et al. (2020). Samples were thawed on ice and centrifuged at 4°C (5 min, 2,000 × g). We mixed 250 μl of saliva with equal parts 0.1% trifluoroacetic acid (TFA)/water, vortexed, and then centrifuged at 4°C (15 min, 17,000 × g). During preliminary tests, we found that this second centrifugation step in TFA helped to separate out additional particles that may have interfered with oxytocin measurement. We loaded the supernatant on 1 ml, 30 mg sorbent, HLB cartridges (Oasis Prime®, cat no. 186008055, Waters Corporation, MA, USA), primed with 1 ml 99% acetonitrile (ACN), followed by 3 ml 0.1% TFA/water. Samples were washed with 3 ml 0.1% TFA/water and eluted with 3 ml 36% ACN/0.1% TFA. Eluents were evaporated using a Vacuum concentrator (SpeedVac® SC210, Thermo Fisher Scientific, MA, USA), and frozen at −80°C until measurement. We measured oxytocin using a commercially available enzyme immuno-assay (EIA) that has been validated for human sOXT (Cayman Chemical ®, kit no. Cay500440, MI, USA). The standard curve ranges from 5.9 to 750 pg ml^−1^ and assay sensitivity is 20 pg ml^−1^. Prior to measurement, we reconstituted samples in 250 μl assay buffer (no dilution with respect to the initial sample volume), and then followed the kit instructions. During a pilot study, we compared a subset of saliva samples collected in duplicate and either first extracted or left unextracted and measured on the same plate. Extracted and unextracted samples were not correlated (r_s_ = 0.36, *n* = 10, *p* = 0.31), suggesting that an extraction step may help to reduce matrix effects. We therefore performed an extraction prior to measurement of sOXT in pigs via enzyme immuno-assay.

We performed validations of parallelism, accuracy/recovery and reproducibility to confirm that the assay measures the majority of oxytocin present and is suitable for measuring oxytocin in pig saliva. To test for parallelism, we estimated and compared the slopes of the fitted lines between the oxytocin standard curve and a serial dilution of pooled pig saliva, using the “lstrends” and “pairs” functions in the package “lsmeans” (Lenth, 2016). The test revealed no differences in the slopes (*t*-test, *n* = 5–6, df = 7, t ratio = −0.002, *p* = 0.999, see Supplementary Figure S1A). Recovery of oxytocin in three different pooled samples of known concentrations, each spiked with oxytocin standards at three different concentrations spanning the linear range (750, 375, 187.5 pg ml^−1^) was 96.4 ± 6.8% (*n* = 9 spiked samples). To determine reproducibility, we measured intra- and inter-assay coefficients of variation (CVs) of pooled low (30 pg ml^−1^) and high (150 pg ml^−1^) control saliva samples that were stored in multiple aliquots, extracted along with test samples and each run in duplicate on each plate. The inter-assay CVs of low- and high-concentration pooled controls were 11.1% and 10.2% respectively (*n* = 28 samples). Intra-assay CVs of low- and high-concentration pooled samples were 13.6% and 10.4% respectively. As an additional analytical validation, we established an immunogram via fractionation with High Performance Liquid Chromatography (HPLC) and measurement with the Cayman EIA, to compare immunoreactivity (IR) in extracted pig saliva and synthetic oxytocin standard containing the complete nanopeptide. We found that 81.9% of the IR detected in the pooled pig saliva sample had the same retention time as the IR in the synthetic oxytocin samples (see Supplementary Figure S2), confirming that our method is suitable for measuring oxytocin in pig saliva. In addition to the analytical validations, we also performed a biological validation by measuring changes in sOXT before and during parturition in *n* = 11 sows. Salivary oxytocin increased almost three-fold during parturition compared to one day before (553.2 ± 372.1 vs. 198.6 ± 102.5 pg ml^−1^, Wilcoxon signed-rank test, *n* = 11, *Z* = −2.41, *p* = 0.014, see Supplementary Figure S3), which is consistent with increases in plasma oxytocin in sows during parturition (Castrén et al., 1993).

For cortisol measurements, we used an EIA kit for salivary cortisol (Demeditec Diagnostics®, GmbH, Kiel, Germany) that has been previously validated in pigs (Goursot et al., 2019). The standard curve ranges from 0.1–30 ng ml^−1^ and assay sensitivity is 0.019 ng ml^−1^. After thawing, samples were spun at 2,500 × g for 5 min, 50 μl of supernatant was diluted 1:2 with assay buffer and samples were run in duplicate. Tests of parallelism revealed no differences in the slopes of the fitted lines between the cortisol standard curve and a serial dilution of pooled pig saliva (*t*-test, *n* = 5–6, df = 7, t ratio = 0.768, *p* = 0.468, see Supplementary Figure S1B). Recovery of sCORT in two different pooled samples of known concentrations, each spiked with cortisol standards at three different concentrations spanning the linear range (30, 7, 1.7 ng ml^−1^) was 100.3 ± 18.3% (*n* = 6 spiked samples). To determine reproducibility, we measured intra- and inter-assay CVs of pooled low (1.5 ng ml^−1^ cortisol) and high (5.0 ng ml^−1^ cortisol) control saliva samples that were stored in multiple aliquots and run in duplicate on each plate. The inter-assay CVs of low- and high-value pooled controls were 9.6% and 8.6% respectively (*n* = 36 samples). Intra-assay CVs of low and high value pooled samples were 10.9% and 8.9% respectively.

### Statistical Analyses

We conducted statistical analyses using the R statistical software (v 4.0.3, R Core Team, 2020). We tested whether social context (baseline, weaning, play, separation or reunion, test predictor) explained variation in sCORT or sOXT concentrations (responses) in pigs. In order to implement the nested, within-subject design of the study, we fit Linear mixed models (LMMs) using the lmer function of the package “lme4” (Bates et al., 2015). We fit two LMMs to estimate sCORT in *n* = 524 samples and sOXT in *n* = 457 samples. We included the age and sex of subjects as additional predictors, to determine whether there were general or sex-specific changes in hormones coinciding with this sensitive developmental period for pigs. We also included predictors for the linear and quadratic time of day of sample collection, to control for linear as well as non-linear effects of time of day on hormone concentrations. We included random intercepts for subjects nested within social group and sow, and for the date of sample collection. To avoid over-fitting the data, and to keep the type I error rate at a nominal 5% (Schielzeth and Forstmeier, 2009; Barr et al., 2013), we also included all theoretically identifiable random slopes (see Supplementary Materials). We log-transformed hormone concentrations to approximate normal distributions, and we z-transformed continuous predictors (age and time of day), to facilitate interpretation of the results.

We confirmed that models were stable by comparing the model estimates derived from the full data set with those obtained from a model with each subject excluded one by one. We also tested for variance inflation using the “vif” function in the package “performance” (Lüdecke et al., 2021) and found no evidence for collinearity of the predictors (VIFs <2.1). We used likelihood ratio tests (Dobson, 2002) to compare each model to a reduced model excluding the effects of social context, age and sex, and present results of models that differed significantly from the reduced model. We tested for significance levels of the fixed effect predictors using *t*-tests with the Satterthwaite approximation (Luke, 2017) in the function lmer of the package lmerTest (v 3.1-3, Kuznetsova et al., 2017) and a model fitted with restricted maximum likelihood. For significant test predictors, we used the “emmeans” package (Lenth, 2021) to perform pair-wise *post-hoc* multiple comparison tests on marginal means and calculated Tukey's HSD-adjusted *p*-values. To confirm that behavioral responses to different social contexts were consistent with the predicted changes in social behavior and general activity levels, we compared agonism, affiliation and activity levels between groups (*n* = 7–8) following the weaning, play and initial baseline observations. We ran Friedmans ANOVAs in the package “exactRankTests” (Hothorn and Hornik, 2021) and conducted *post-hoc* Wilcoxon tests with Bonferroni corrections for multiple testing, using the package “rstatix” (Kassambara, 2021). We also compared frequencies of agonistic and affiliative interactions between reunited pigs (*n* = 74) and their group members in the 15-min following reunions. Results are presented as mean (± SD). We tested for significance using estimated marginal means (± SE) based on the fitted statistical model, adjusting for the fixed and random effects in the model. A *p*-value <0.05 was considered a significant effect.

## Results

**Figure 2:**
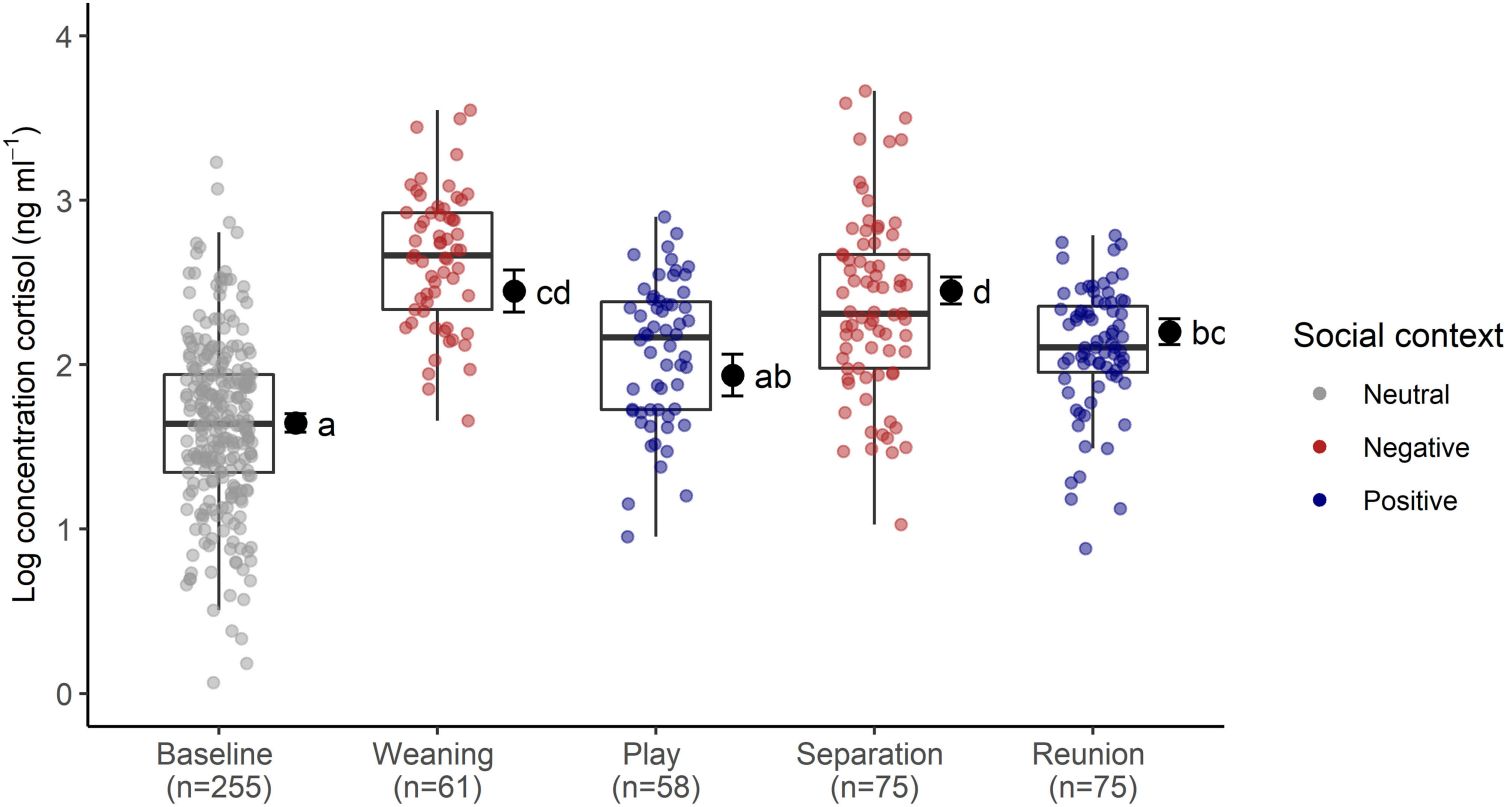
Log transformed salivary cortisol concentrations following neutral, negative and positively-valenced social contexts. Boxplots indicate medians and interquartile ranges. Sample size for each context is also indicated. Results of the pair-wise *post-hoc* multiple comparisons (Tukey's HSD) are visualized using a compact letter display, with different letters indicating significant differences. For full model results, see Table 2.

The full cortisol model differed significantly from a reduced model that did not include the main effects of social context, age and sex (χ^2^ = 85.56, *df* = 6, *p* < 0.001). Social context had a strong effect on sCORT concentrations [*F*_(4, 23)_ = 54.07, *p* < 0.001, Table 1; Figure 2]. There were up to three-fold increases in sCORT following negative social challenges (weaning: 15.22 ± 6.3 ng ml^−1^), separation: 12.32 ± 7.49 ng ml^−1^), compared to baseline (5.87 ± 3.28 ng ml^−1^, both *p* values <0.001). Considering positive social challenges, sCORT following play did not differ from baseline (*p* = 0.233). Post-reunion sCORT (8.71 ± 2.97 ng ml^−1^) remained elevated compared to baseline concentrations (*p* < 0.001), although sCORT was lower following reunions than during separations (*p* = 0.019). Pigs also had decreases in sCORT with increasing age [F_(1, 31)_ = 4.68, *p* = 0.038, Table 1]. Cortisol concentrations were not influenced by subjects' sex or the time of sample collection (see Table 1).

**Table 1:** Results of a linear mixed model testing for effects of social context on salivary cortisol concentrations in young pigs.

	**Estimate**	**SE**	***df***	***t* value**	***p***
(Intercept)	1.655	0.061	56.36	26.977	
Context—Weaning^a^	0.802	0.137	26.80	5.84	**<0.001**
Context—Play^a^	0.291	0.133	15.631	2.184	**0.045**
Context—Separation^a^	0.805	0.069	10.891	11.684	**<0.001**
Context—Reunion^a^	0.555	0.063	394.276	8.76	**<0.001**
Time sample collection	−0.011	0.035	64.524	−0.307	0.760
Polynomial time sample collection	−0.015	0.024	257.178	−0.633	0.527
Age	−0.118	0.055	31.158	−2.163	**0.038**
Sex—Male^b^	0.005	0.058	20.908	0.092	0.928

*^a, b^Estimates refer to comparison with the reference categories.*

*^a^Context—Baseline.*

*^b^Sex—Female.*

*P-values are estimated based on t-tests using the Satterthwaite approximation to degrees of freedom. Significant effects are indicated in bold*.

**Figure 3:**
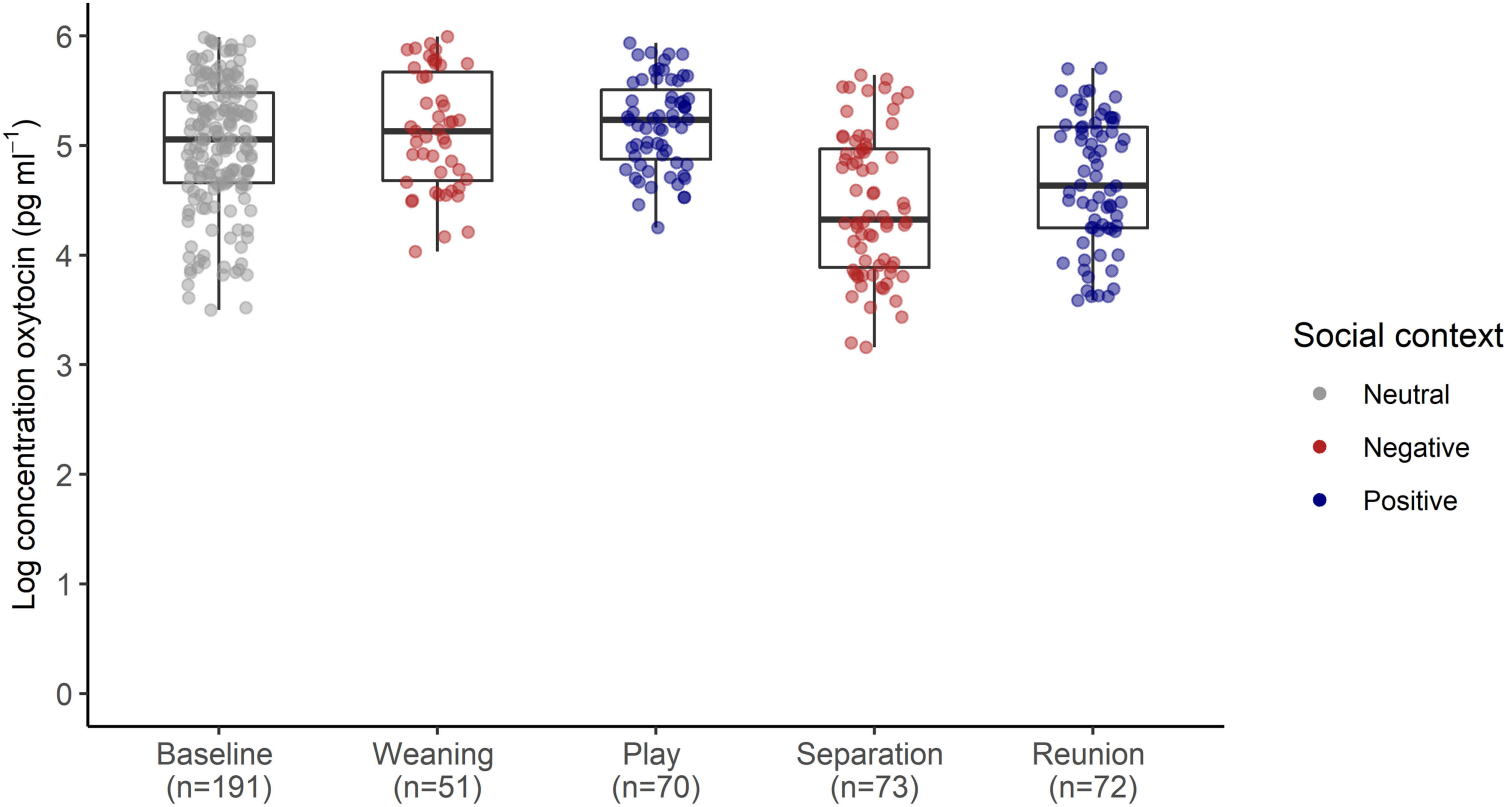
Log transformed salivary oxytocin concentrations following neutral, negative and positively-valenced social contexts. Boxplots indicate medians and interquartile ranges. Sample size for each context is also indicated. For full model results, see Table 2.

The full oxytocin model differed significantly from a reduced model with the main effects of social context, age and sex excluded (χ^2^ = 15.57, *df* = 6, *p* = 0.016). In contrast with predictions, sOXT concentrations did not differ between the different social contexts [*F*_(4, 12)_ = 1.66, *p* = 0.22, Table 2; Figure 3]. Rather than increasing in response to positive social challenges, sOXT concentrations were at their lowest during a brief social isolation (see Figure 3), although this difference did not reach significance. Regardless of context, pigs had lower sOXT with increasing age [*F*_(1, 24)_ = 8.54, *p* = 0.007, see Table 2]. The other control predictors did not influence sOXT.

**Table 2:** Results of a linear mixed model testing for effects of social context on salivary oxytocin concentrations in young pigs.

	**Estimate**	**SE**	***df***	***t* value**	***P***
(Intercept)	5.044	0.110	12.245	45.912	
Context—Weaning^a^	−0.23	0.231	24.114	−0.993	0.331
Context—Play^a^	0.007	0.191	18.620	0.039	0.97
Context—Separation^a^	−0.243	0.107	7.576	−2.272	0.054
Context—Reunion^a^	−0.020	0.071	9.227	−0.282	0.784
Time of sample collection	−0.079	0.043	41.481	−1.854	0.071
Polynomial time of collection	0.032	0.026	170.090	1.235	0.219
Age	−0.229	0.078	24.003	−2.923	**0.007**
Sex- Male^b^	−0.041	0.067	18.623	−0.609	0.55

*^a, b^Estimates refer to comparison with the reference categories.*

*^a^Context—Baseline.*

*^b^Sex—Female.*

*P-values are estimated based on t-tests using the Satterthwaite approximation to degrees of freedom. Significant effects are indicated in bold*.

**Figure 4:**
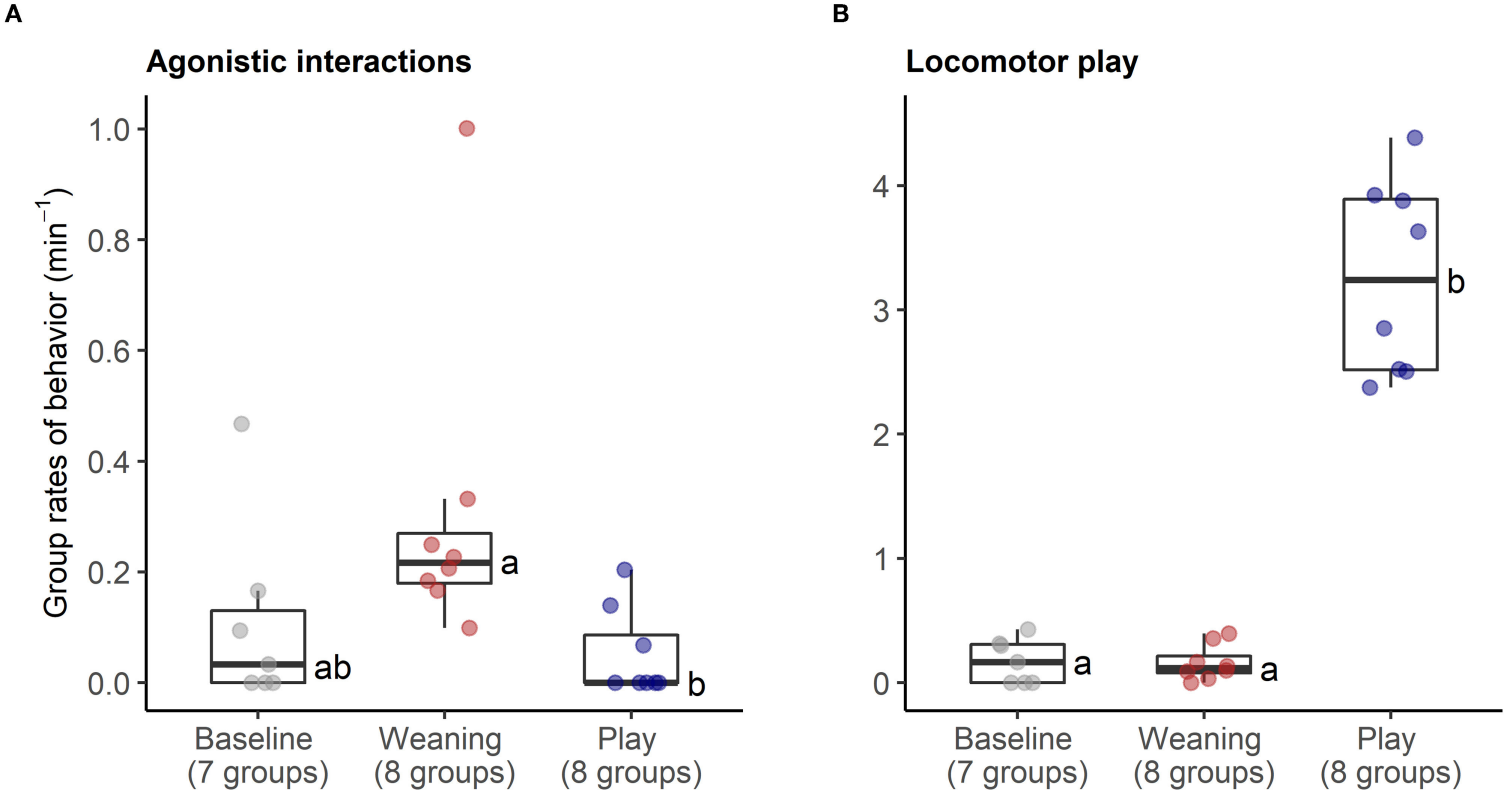
Rates of agonistic interactions **(A)** and locomotor play **(B)** within social groups during neutral (baseline), negative (weaning) and positive (play) social contexts. The number of social groups sampled in each context is indicated. Boxplots indicate medians and interquartile ranges. Results of the pair-wise *post-hoc* multiple comparisons (Bonferroni tests) are visualized using a compact letter display, with different letters indicating significant differences.

**Figure 5:**
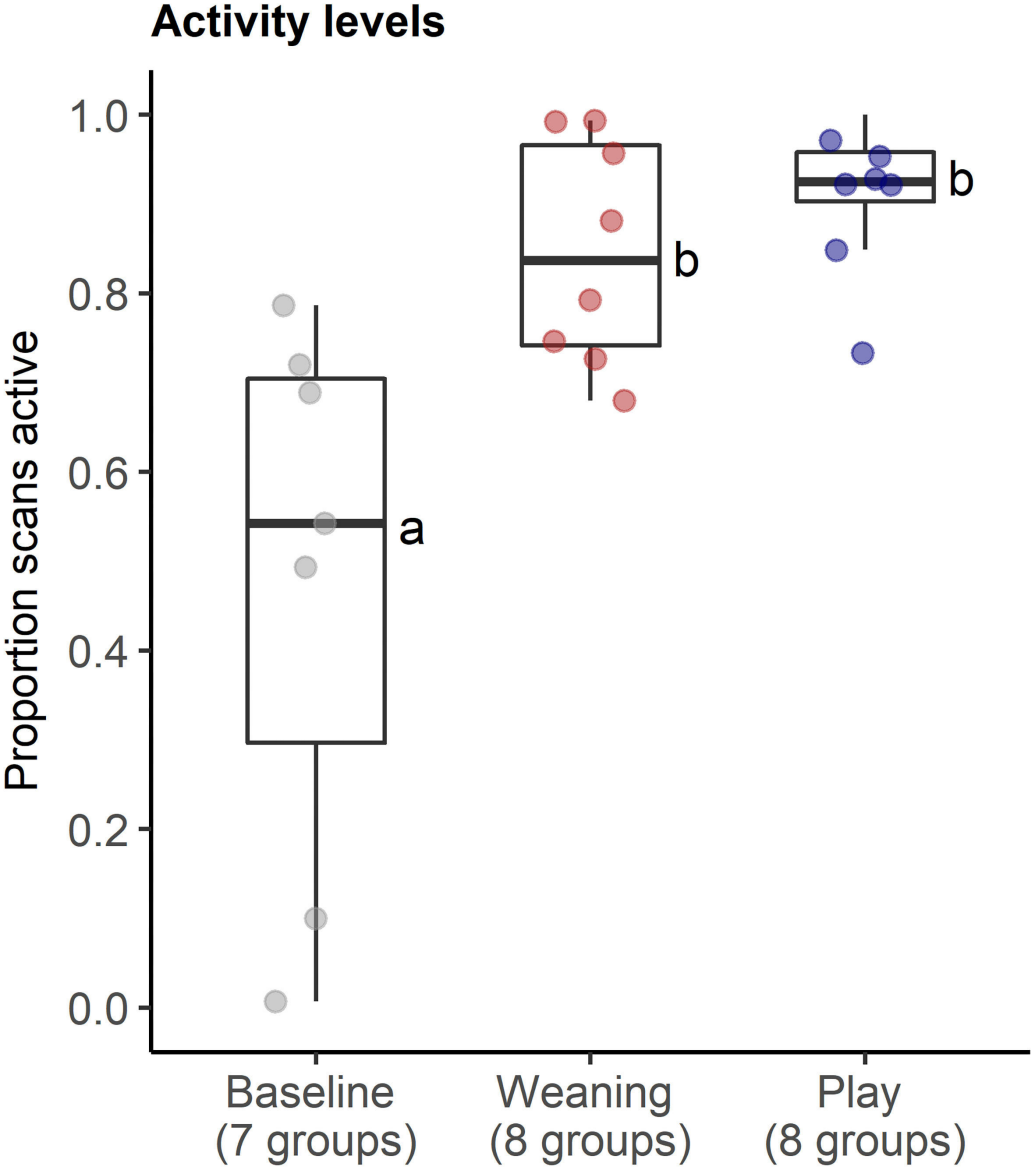
Proportion of instantaneous scans during which pigs were active in neutral (baseline), negative (weaning) and positive (play) social contexts. The number of social groups sampled in each context is indicated. Results of the pair-wise *post-hoc* multiple comparisons (Bonferroni tests) are visualized using a compact letter display, with different letters indicating significant differences.

The behavioral analyses confirmed our predictions about the expected changes in social interactions related to the different social challenges. Groups differed in frequencies of agonistic interactions (Friedmans, χ^2^ = 9.54, *df* = 2, *p* = 0.008), due to increases in agonism following weaning (0.31 ± 0.29 events per minute) compared with following play (0.05 ± 0.08 agonistic events per minute, Wilcoxon, p.adj = 0.023, Figure 4A). Post-weaning agonism did not differ from baseline (0.11 ± 0.17 events per minute, Wilcoxon, p.adj = 0.657, see Figure 4A). Groups also differed in frequencies of locomotor play across the different contexts (Friedmans, χ^2^ = 10.89, *df* =2, *p* = 0.004), due to increases in locomotor play when pigs had free access to a large hallway (3.26 ± 0.78 events per minute), in comparison with locomotor play during weaning (0.16 ± 0.14 events per minute, Wilcoxon, p.adj = 0.023), and baseline (0.17 ± 0.18 events per minute, Wilcoxon, p.adj = 0.047, Figure 4B). The effects of the play context on group locomotor play were especially evident, as this synchronized play occurred 0.87 ± 0.23 times per minute during the play condition, and often involved the majority of individuals in the group (see Supplementary Video S1). In contrast, group locomotor play was rare in other contexts, occurring only once during the weaning observations, and only seven times during the baseline observations. Groups also tended to have more affiliative interactions during play than in the other social contexts (Friedmans, χ^2^ = 5.42, *df* = 2, *p* = 0.06), although *post-hoc* pairwise comparisons were not significant (p.adj > 0.12). Differences in activity levels among the different contexts (Friedmans, χ^2^ = 11.14, *df* = 2, *p* = 0.003) were also consistent with increased energetic expenditure during negative and positive social challenges. Pigs were standing or moving more often during weaning (84.6 ± 12.6% of scans, Wilcoxon, p.adj = 0.047) and play (91 ± 8.4% of scans, Wilcoxon, p.adj = 0.047), in comparison with baseline contexts (47.7 ± 30.8% of scans, Figure 5). Activity levels did not differ between the weaning and play conditions (Wilcoxon, p.adj = 0.75, see Figure 5).

**Figure 6:**
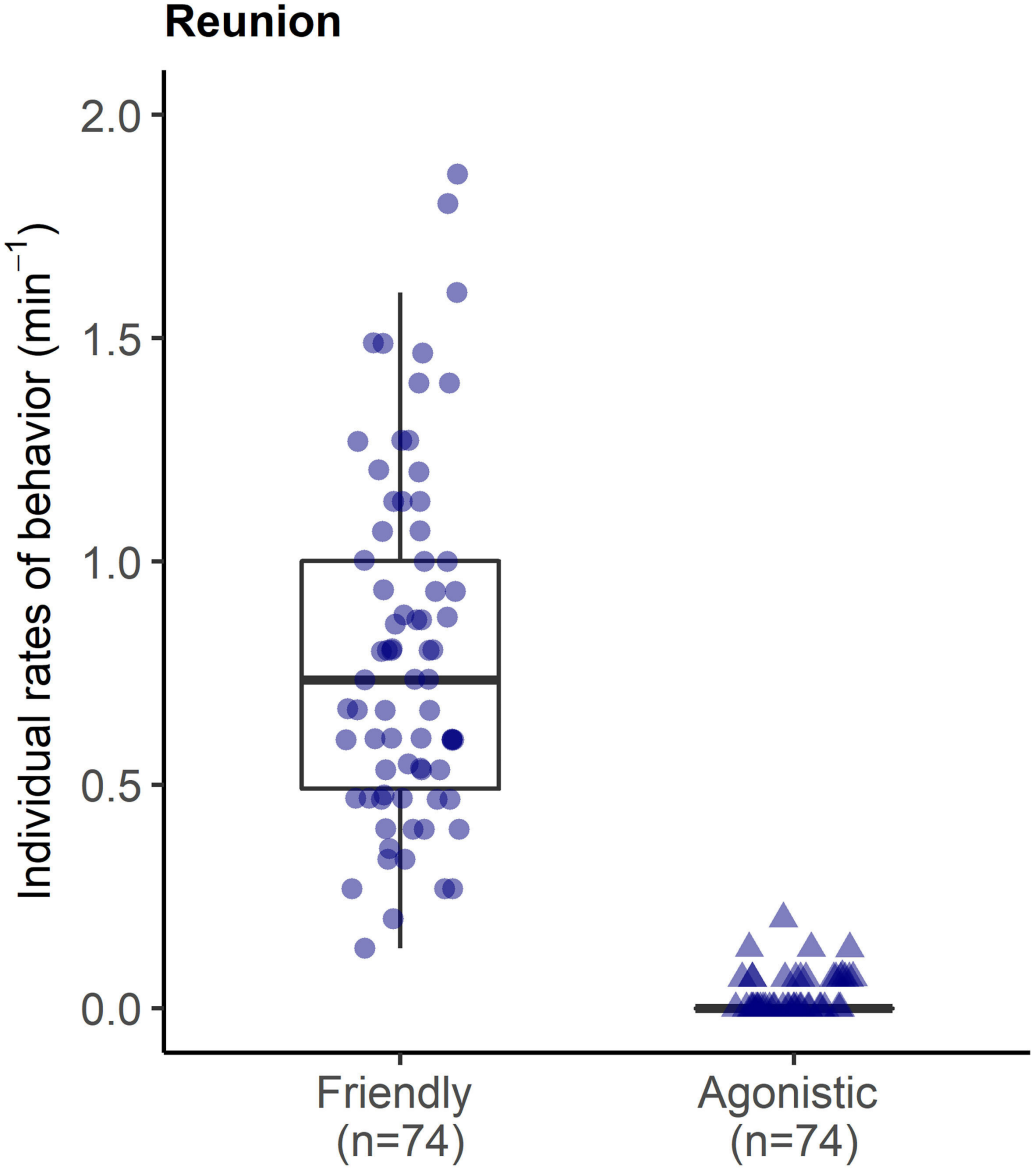
Rates of affiliative and agonistic interactions between each reunited pig and their group members in the 15 min reunion period after a brief social isolation.

During reunions after separations, reunited pigs were involved in high rates of affiliative interactions (0.80 ± 0.39 events per min), including prolonged bouts of licking/nibbling behavior (see Supplementary Video S2), and low rates of agonism (0.02 ± 0.04 per min) with other group members (Figure 6).

## Discussion

We compared sCORT and sOXT concentrations in pigs under neutral social conditions and following changes in social contexts that were expected to be negatively- or positively-valenced. Our goals were to examine the interplay between changes in sCORT and sOXT in response to social challenges, and to evaluate the usefulness of combining these hormones as indicators of the intensity and valence of emotional responses. As expected, pigs had the highest sCORT concentrations following exposure to involuntary social challenges that posed potential threats to fitness. Voluntary, positive social challenges were also associated with increased sCORT concentrations relative to baseline, although these differences were only significant for the reunion context. These results are consistent with evidence in other species that peripheral cortisol release can be associated with energetically-demanding positive challenges (e.g. voluntary exercise: Hew-Butler et al., 2008, sexual arousal: Buwalda et al., 2012), and caution against using glucocorticoid responses alone as indicators of emotional valence or for welfare purposes (Veissier and Boissy, 2007). Our results also support the hypothesis that relative cortisol reactivity to different contexts may reflect the intensity of emotional responses, whereby a combination of psychosocial and energetic stressors (such as during weaning) may trigger greater cortisol reactivity than energetic stress alone (such as during play). Reimert et al. (2013) also found graded increases in sCORT in pigs exposed to positive (access to environmental enrichment) or negative (brief isolation and aversive interactions with humans) contexts, with the strongest responses to the negative stimuli.

In contrast with our assumptions, sOXT concentrations following positive and negative social challenges did not differ consistently from baseline. These results contradict studies in other species reporting increases in peripheral oxytocin in response to stressors that are proportional to the strength of the glucocorticoid stress response (e.g. humans: Hew-Butler et al., 2008; Engert et al., 2016; rodents: Neumann et al., 1998, 2001a). However, our results are consistent with other studies that also report no changes in peripheral oxytocin in response to stressors (Altemus et al., 2001; Taylor et al., 2006; Ditzen et al., 2007; McQuaid et al., 2016). Some of the variation in these results likely relates to context-specific features of stressors that may have different influences on the oxytocinergic system. However, there are also contradictory findings regarding peripheral oxytocin release in response to the same stressor (e.g. the Trier Social Stress Test: Altemus et al., 2001; Pierrehumbert et al., 2012; Engert et al., 2016). In addition, studies sometimes measure the two hormones in different matrices (e.g. Ditzen et al., 2007; Seltzer et al., 2010; Pierrehumbert et al., 2012; Engert et al., 2016) and/or at different time frames following the stressor (e.g. Neumann et al., 1998; Pierrehumbert et al., 2012; Engert et al., 2016), making it difficult to compare hormonal responses within and between studies. Future studies should measure these hormones in the same matrices and at multiple time points before, during and after exposure to a range of differently-valenced challenges, to clarify the time course for endogenous release of oxytocin and cortisol. In addition, manipulating access to social support during stressors and examining how this influences the timing and extent of peripheral oxytocin and cortisol release will help to further clarify the role of the oxytocinergic system in social buffering from stressors.

It is interesting that pigs exhibited their lowest sOXT concentrations following a brief period of social isolation, since this was the only context in which pigs had no access to social support. In contrast, during weaning as well as under baseline conditions, pigs had familiar littermates present who could provide social support. In other species, short-term social isolation is associated with alterations in the central oxytocinergic system (rats: Oliveira et al., 2019) and decreases in peripheral oxytocin (rats: Harvey et al., 2019, guinea-pigs: Machatschke et al., 2004). Furthermore, oxytocin concentrations tended to increase during reunions, when reunited pigs were involved in prolonged affiliative tactile contact that has been linked to the oxytocinergic system in other species (Dunbar, 2010). It would be interesting to test whether contexts that promote increased affiliative interactions in pigs without first requiring the removal of social contact may lead to increased sOXT concentrations relative to baseline. In one relevant study, there were no differences in sOXT concentrations when pigs were separated from their social groups without any social contact, or with opportunities for affiliative contact with humans (Lürzel et al., 2020). However, during the affiliative contact condition, sOXT was positively correlated with the amount of affiliative stroking that pigs received (Lürzel et al., 2020).

Young pigs given short-term access to a hallway that increased their total available space exhibited increased activity levels, including exaggerated movements such as scampering and pivoting that serve no apparent functional purpose, which is one hallmark of play behavior (Held and Špinka, 2011). The play context also triggered the expected increases in synchronized group movement, which may help to enhance security and group cohesion in pigs (Špinka and Marek, 2012). Other studies reporting a link between behavioral synchrony and peripheral oxytocin have typically focused on interactions between parents and offspring (Feldman, 2012) or between in-group members in response to out-group threats (De Dreu, 2012; Samuni et al., 2017; Jiang and Platt, 2018), but have rarely looked at behavioral coordination in the context of play. The few studies to investigate oxytocin during play do not support a direct role of oxytocin in mediating play behavior. Mini-pigs (*Sus scrofa domesticus*) given opportunities for novel object play had significantly *lower* plasma oxytocin concentrations afterwards in comparison with control pigs that did not have access to play opportunities (Marcet Rius et al., 2018). In rats, exogenously-administered oxytocin either increased, decreased or had no effect on social play depending on the sex of subjects and the context in which play occurred (Bredewold et al., 2014). Given that play behavior is considered a hallmark of positive welfare in young pigs (Lawrence et al., 2018) and in captive animals more generally, identifying biomarkers that are altered during play behavior would help to advance research on positive emotions in animals.

Pigs exhibited a high degree of individual variation in physiological measurements, under baseline conditions as well as after exposure to social challenges. Our model results indicate that some of the variation in cortisol and oxytocin measurements was related to developmental changes in these young pigs during the study period. Other studies indicate similar reductions in sCORT in growing pigs with age, until around 20 weeks of age when more stable adult circadian rhythms are reached (Ruis et al., 1997). Less is known about the development of the oxytocinergic system in pigs, but there are similar decreases in peripheral oxytocin concentrations during development in human infants and children (Nishizato et al., 2017) and in young rats (Elabd et al., 2014). Based on evidence in humans (Bartz et al., 2011; Shamay-Tsoory and Abu-Akel, 2016), it is likely that a range of other factors, including personality, social dominance and previous experience may have affected behavioral and hormonal responses of individuals to changes in social context, and these themes will be explored in follow-up studies.

Salivary free cortisol is a reliable measure of the biologically active faction of cortisol in animals (Hellhammer et al., 2009), and pigs exhibit correlated increases in cortisol in plasma and saliva within minutes in response to an ACTH challenge (Bushong et al., 2000). Salivary oxytocin is increasingly being favored as a non-invasive alternative to more established methods that rely on cerebrospinal fluid or blood (reviewed in Valstad et al., 2017). Valid concerns remain about inconsistencies in laboratory methods for oxytocin measurement, especially regarding the need for extractions, which may vary depending on the species, matrix and analytical technique (reviewed in MacLean et al., 2019). There is also a lack of knowledge of the mode of transfer of OXT from the blood to saliva (Gröschl, 2009). These concerns can be addressed in part through thorough analytical and biological validations of sOXT, which have now been conducted in several species (dogs: MacLean et al., 2018, pigs: López-Arjona et al., 2020a, humans: de Jong et al., 2015), and also by companies producing commercially-available OXT EIA kits (Cayman Chemical®, MI, USA). Although our laboratory methods differ from those of López-Arjona et al. (2020a), our biological validation confirmed significant increases in sOXT during parturition in sows, consistent with their findings. Furthermore, the results of our immunogram confirm that the majority of OXT detected in extracted saliva samples has properties that are consistent with oxytocin standards containing the complete nonapeptide. However, even when accurately measured, peripheral oxytocin concentrations do not always reflect central oxytocinergic system activation. For example, rats experiencing a stressor due to social defeat exhibited increases in central oxytocin, measured by intracerebral microdialysis, but had decreased plasma oxytocin concentrations (Engelmann et al., 2001; Neumann, 2002). Thus our results indicate only that sOXT concentrations under varied social challenges were not related to changes in sCORT, or to rates of agonistic, affiliative or play behavior within groups. We conclude that current methods can detect changes in sOXT in pigs related to biological processes that are mediated by the oxytocinergic system such as parturition (López-Arjona et al., 2020a, this study) and ejaculation (López-Arjona et al., 2020b), but that there is as yet not convincing evidence that acute changes in sOXT reflect positively- or negatively-valenced social contexts, or underlying emotional states, in pigs (Rault et al., 2017; Lürzel et al., 2020, this study).

Our sampling time points were meant to capture short-term changes in sCORT and sOXT coinciding with responses to social challenges, based in part on a meta-analysis in humans suggesting that peripheral oxytocin and cortisol are more likely to be correlated soon after the onset of various challenges, while later measurements may be influenced by inhibitory feedback between the two systems (Brown et al., 2016). Both cortisol and oxytocin have short half-lives in plasma, and in a range of species, increases in sCORT or sOXT are detectable within 10–15 min following exposure to relevant stimuli (e.g. de Jong et al., 2015; MacLean et al., 2018). We therefore assumed that sampling 15 min following separations and reunions would capture physiological responses to the abrupt changes in the social environment. We chose longer time frames of 30 min following initial exposure to the play context and 60 min following weaning, since we expected delays before pigs exhibited increases in the relevant social behaviors. Our behavioral analyses confirmed that the time frames immediately prior to saliva sampling captured increases in the social behaviors of interest, and sCORT concentrations indicate that pigs were experiencing HPA-axis activation 60 min following weaning. However, it is possible that we failed to detect shorter-term changes in peripheral oxytocin following play and weaning, due to the delayed sampling time frames. It would be interesting to collect sOXT in pigs soon after the onset of other negative social stressors, such as weaning, to test whether sOXT concentrations may show initial decreases and then a return to baseline, similar to the tendencies observed shortly after separations and reunions.

Understanding how the HPA axis interacts with other biological systems, including the oxytocinergic system, will help to evaluate how different challenges are experienced by individuals, and how social support may influence stress reactivity (Brown et al., 2016). Research into the interactions between the HPA axis and the oxytocinergic system can be facilitated by experimental manipulation of these systems, for example through exogenous administration (e.g. Cardoso et al., 2013) or through the use of antagonists (e.g. Neumann et al., 2000). It is also important to understand the causes and effects of endogenous changes in these hormones in different species in response to biologically-relevant challenges. This research is being aided by an increasing tool-kit of non-invasive methods to measure peripheral hormones in a range of matrices (Kremer et al., 2020). We found that pigs exposed to differently-valenced social challenges showed graded increases in sCORT, suggesting that cortisol may indicate the intensity of emotional responses. We did not find variation in sOXT in response to challenges, although sOXT tended to decrease upon removal of social support, and to increase again when social support was restored. Further research characterizing peripheral oxytocin responses to biologically-relevant challenges with and without access to social support will help to clarify the role of the oxytocinergic system in mediating HPA-axis reactivity to challenges.

## Data Availability Statement

The datasets presented in this study can be found in online repositories. The names of the repository/repositories and accession number(s) can be found online at: https://doi.org/10.5281/zenodo.6510974.

## Ethics Statement

The animal study was reviewed and approved by the Committee for Animal Use and Care of the Agricultural Department of Mecklenburg-Western Pomerania, Germany (permit LALLF 7221.3-1-036/20).

## Author Contributions

LRM and UG contributed to conception and design of the study. LRM collected the data and wrote the first draft of the manuscript. LRM and WO designed and optimized the HPLC validation. LRM and AE performed the statistical analysis. AE created the figures and the Docker container. All authors contributed to manuscript revision, read, and approved the submitted version.

## Funding

The authors are grateful for funding from the Universities Federation for Animal Welfare (UFAW, Grant No. 44 19/20 to LRM) in support of this research. The publication of this article was partially funded by the Open Access Fund of the FBN.

## Conflict of Interest

The authors declare that the research was conducted in the absence of any commercial or financial relationships that could be construed as a potential conflict of interest.

## Publisher's Note

All claims expressed in this article are solely those of the authors and do not necessarily represent those of their affiliated organizations, or those of the publisher, the editors and the reviewers. Any product that may be evaluated in this article, or claim that may be made by its manufacturer, is not guaranteed or endorsed by the publisher.
