# Supplementary material for: Salivary Cortisol, but Not Oxytocin, Varies With Social Challenges in Domestic Pigs: Implications for Measuring Emotions
Source: Front Behav Neurosci. 2022 May 23;16:899397. doi: 10.3389/fnbeh.2022.899397 (PMC9169876; doi:10.3389/fnbeh.2022.899397)
Supplement: Supplementary file 1 [file Table_1.DOCX]

Supplementary Material: Cortisol, oxytocin and social challenges in pigs. L. R. Moscovice, U. Gimsa, W. Otten & A. Eggert

# Supplementary data

## Power analyses

Power analyses were performed for repeated measures ANOVAs, using the ‘WebPower’ package (Zhang & Mai, 2018) in R (v. 4.0.5); (<https://rdrr.io/cran/WebPower/man/wp.rmanova.html>).

Explanation of abbreviations in code:

- Ng = number of groups or number of levels of the between-subject factor that are used in the study design. We indicated three groups, representing the baseline, negative and positive social contexts
- Nm = number of measurements, referring to the number of different measurements per subject. We indicated two measures, referring to two samples in each social context.
- F = Cohens f estimate of effect size. We gave a Cohens f estimate for detecting changes in salivary cortisol following social stressors of 0.32, based on a meta-analysis by Michaud and colleagues 2008 (Table 3, converted from Cohens d value= 0.63). We give a Cohens f estimate for detecting changes in salivary oxytocin following stressors of 0.34, based on a meta-analysis by Brown and colleagues 2016 (Fig 1, converted from a Pearsons r value= 0.32).
- Nscor = nonsphericity coefficient, estimated to be 1.0
- Type = 1 (indicates a within-effect test about the mean difference among measurements)
- Power curve for part a) Detecting salivary cortisol responses to stressors
- library(WebPower)
  wp.rmanova(n=seq(50,90,4), ng=3, nm=2, f=0.32, nscor=1.0, type=1)
- ## Repeated-measures ANOVA analysis
  ##
  ## n f ng nm nscor alpha power
  ## 50 0.32 3 2 1 0.05 0.6011857
  ## 54 0.32 3 2 1 0.05 0.6357500
  ## 58 0.32 3 2 1 0.05 0.6679914
  ## 62 0.32 3 2 1 0.05 0.6979598
  ## 66 0.32 3 2 1 0.05 0.7257242
  ## 70 0.32 3 2 1 0.05 0.7513687
  ## 74 0.32 3 2 1 0.05 0.7749885
  ## 78 0.32 3 2 1 0.05 0.7966861
  ## 82 0.32 3 2 1 0.05 0.8165688
  ## 86 0.32 3 2 1 0.05 0.8347463
  ## 90 0.32 3 2 1 0.05 0.8513287
  ##
  ## NOTE: Power analysis for within-effect test
  ## URL: http://psychstat.org/rmanova


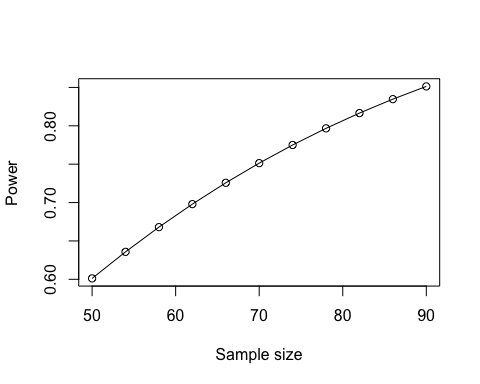


Results suggest that a sample size of n = 78 pigs should be sufficient to detect a true effect with 80% probability

Power curve for part b) Detecting salivary oxytocin responses to stressors

library(WebPower)
wp.rmanova(n=seq(50,90,4), ng=3, nm=2, f=0.34, nscor=1.0, type=1)

## Repeated-measures ANOVA analysis
##
## n f ng nm nscor alpha power
## 50 0.34 3 2 1 0.05 0.6535323
## 54 0.34 3 2 1 0.05 0.6883778
## 58 0.34 3 2 1 0.05 0.7203798
## 62 0.34 3 2 1 0.05 0.7496548
## 66 0.34 3 2 1 0.05 0.7763384
## 70 0.34 3 2 1 0.05 0.8005786
## 74 0.34 3 2 1 0.05 0.8225305
## 78 0.34 3 2 1 0.05 0.8423527
## 82 0.34 3 2 1 0.05 0.8602032
## 86 0.34 3 2 1 0.05 0.8762373
## 90 0.34 3 2 1 0.05 0.8906055
##
## NOTE: Power analysis for within-effect test
## URL: http://psychstat.org/rmanova


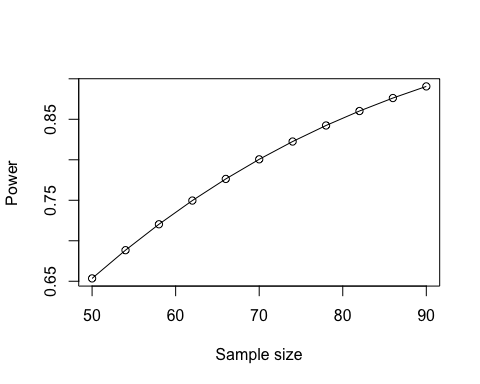


Results suggest that a sample size of n = 78 pigs should be sufficient to detect a true effect with 84% probability

References

Brown, C. A., Cardoso, C., and Ellenbogen, M. A. (2016). A meta-analytic review of the correlation between peripheral oxytocin and cortisol concentrations. *Front. Neuroendocrinol*. 43, 19–27. <doi:10.1016/j.yfrne.2016.11.001>

Michaud, K., Matheson, K., Kelly, O., and Anisman, H. (2008). Impact of stressors in a natural context on release of cortisol in healthy adult humans: A meta-analysis. *Stress* 11, 177–197. <doi:10.1080/10253890701727874>

Zhiyong Zhang and Yujiao Mai (2018). WebPower: Basic and Advanced Statistical Power Analysis. R package version 0.5.2. <https://CRAN.R-project.org/package=WebPower>

**1.2 Supplementary statistical information: Determination of random effects structure**

It is not trivial to decide which random intercept and random slopes structures to include in mixed models. We used the function fe.re.tab() written by Roger Mundry that facilitates decisions based on the following two rules:

Rule 1: We include a random slope of a covariate when there are at least three unique values of the covariate per level of the random effect (otherwise the random slope would be unidentifiable)

Rule 2: We include a random slope of a factor when there are at least two observations per level of the random effect (otherwise the random slope would be unidentifiable)

We include random slopes if these requirements are fulfilled for at least half of the levels of the random effect. The function fe.re.tab() determines the number of levels of the random effect per number of unique cases of the fixed effect with at least two observations.

Results for cortisol model:

## $`age.z_within_uniq.id (matrix)`

## 2 3 4 5 6 7 8 tot

## 3 12 12 33 10 5 3 78

##

## $`age.z_within_sow (matrix)`

## 6 8 9 10 11 12 13 tot

## 1 1 2 1 4 6 2 17

##

## $`age.z_within_uniq.group (matrix)`

## 10 11 13 14 tot

## 1 1 2 4 8

##

## $`age.z_within_date (matrix)`

## 1 2 tot

## 50 1 51

##

## $`context_within_uniq.id (factor)`

## 0 1 tot

## 3 75 78

##

## $`context_within_sow (factor)`

## 3 4 5 tot

## 1 2 14 17

##

## $`context_within_uniq.group (factor)`

## 5 tot

## 8 8

##

## $`context_within_date (factor)`

## 1 3 tot

## 33 18 51

##

## $`sex_within_uniq.id (factor)`

## 1 tot

## 78 78

##

## $`sex_within_sow (factor)`

## 2 tot

## 17 17

##

## $`sex_within_uniq.group (factor)`

## 2 tot

## 8 8

##

## $`sex_within_date (factor)`

## 1 2 tot

## 8 43 51

##

## $`time.n.z_within_uniq.id (matrix)`

## 1 2 3 4 5 6 7 8 9 10 tot

## 1 1 1 2 11 18 27 11 5 1 78

##

## $`time.n.z_within_sow (matrix)`

## 12 14 15 17 18 20 25 26 28 29 30 31 32 47 tot

## 1 1 1 1 1 1 1 1 2 1 3 1 1 1 17

##

## $`time.n.z_within_uniq.group (matrix)`

## 38 39 46 47 50 55 56 tot

## 1 1 2 1 1 1 1 8

##

## $`time.n.z_within_date (matrix)`

## 1 2 3 4 7 8 9 10 11 12 13 14 15 16 tot

## 1 4 12 2 2 1 8 5 4 4 1 1 4 2 51

Results for oxytocin model:

## $`age.z_within_uniq.id (matrix)`

## 1 2 3 4 5 6 7 tot

## 2 3 16 33 20 3 1 78

##

## $`age.z_within_sow (matrix)`

## 5 6 7 8 9 10 11 tot

## 1 1 2 4 3 2 4 17

##

## $`age.z_within_uniq.group (matrix)`

## 10 11 12 13 14 tot

## 2 2 2 1 1 8

##

## $`age.z_within_date (matrix)`

## 1 tot

## 52 52

##

## $`context_within_uniq.id (factor)`

## 0 1 tot

## 8 70 78

##

## $`context_within_sow (factor)`

## 3 5 tot

## 1 16 17

##

## $`context_within_uniq.group (factor)`

## 5 tot

## 8 8

##

## $`context_within_date (factor)`

## 0 1 3 tot

## 7 27 18 52

##

## $`sex_within_uniq.id (factor)`

## 0 1 tot

## 1 77 78

##

## $`sex_within_sow (factor)`

## 1 2 tot

## 1 16 17

##

## $`sex_within_uniq.group (factor)`

## 2 tot

## 8 8

##

## $`sex_within_date (factor)`

## 0 1 2 tot

## 9 14 29 52

##

## $`time.n.z_within_uniq.id (matrix)`

## 1 2 3 4 5 6 7 8 tot

## 1 1 5 2 18 29 19 3 78

##

## $`time.n.z_within_sow (matrix)`

## 12 13 16 17 18 22 23 24 25 26 27 29 30 39 tot

## 1 1 1 1 1 1 1 2 2 1 1 2 1 1 17

##

## $`time.n.z_within_uniq.group (matrix)`

## 39 40 41 42 45 46 47 54 tot

## 1 1 1 1 1 1 1 1 8

##

## $`time.n.z_within_date (matrix)`

## 1 2 3 4 7 8 9 10 11 12 13 14 15 18 20 tot

## 8 10 3 1 1 4 6 1 5 3 3 2 3 1 1 52

Based on the results of the helper function fe.re.tab(), we defined the following identical structure of the linear mixed models for both cortisol and oxytocin:

- random intercepts + random slopes for these fixed effects within date
  - INCLUDE: sex, time.n.z
  - EXCLUDE: context, age.z
- random intercepts + random slopes for these fixed effects within sow
  - INCLUDE: context, age.z, sex, time.n.z
  - EXCLUDE: *none*
- random intercepts + random slopes for these fixed effects within uniq.group
  - INCLUDE: context, age.z, sex, time.n.z
  - EXCLUDE: *none*
- random intercepts + random slopes for these fixed effects within uniq.id
  - INCLUDE: age.z, time.n.z
  - EXCLUDE: context, sex

**1.3 Supplementary laboratory validations of salivary oxytocin measurement in pigs**

As an additional analytical validation, we established an immunogram via fractionation with High Performance Liquid Chromatography (HPLC) to compare immunoreactivity in pig saliva and synthetic OXT standard. We first injected 100 µl ultra-pure water with 100 ng of pure oxytocin containing the complete nonapeptide (Sigma-Aldrich, Taufkirchen, Germany, No. O6379) into the HPLC system (LC-20, SHIMADZU Deutschland, Duisburg, Germany), equipped with a 250 x 4 mm column packed with Prontosil C18 AQ (Bischoff Analysentechnik, Leonberg, Germany). The sample was run in a mobile phase with 22% ACN/0.1% TFA at a flow rate of 1 ml min^-1^. We determined via chromatogram that pure oxytocin elutes after 6.8 min (see Supplementary Figure S2a). We then extracted 500 µl of 1) a pooled pig saliva sample and 2) an oxytocin standard sample (750 pg ml^-1^) that was prepared by adding 50 µl of a 7.5 ng ml^-1^ concentration of pure oxytocin standard containing the complete nonapeptide (Cayman chemicals, MI, USA, CAS Number 50-56-6) to 450 µl assay buffer. We extracted both samples following the protocol detailed in the manuscript, adjusted for 500 µl extraction volumes. After evaporation, samples were frozen in -80º C until run via HPLC. In preparation for HPLC, samples were thawed and resuspended in ultra-pure water (100 µl for the pooled saliva sample to concentrate the sample, and 300 µl for the oxytocin standard). Samples were vortexed and 100 µl were injected into the HPLC system and run using the same conditions as described above. We collected eight fractions, with 3 ml per fraction, starting 1 min before injection. We evaporated fractions using a SpeedVac and froze them at -80º C until measurement via the Cayman enzyme immune-assay as described in the manuscript, except that samples were resuspended in 500 µl assay buffer (to maintain a 1:1 ratio).

We determined the immunoreactivity (IR) in each fraction obtained from the HPLC based on the EIA plate measurements. We first determined in which of the oxytocin standard fractions IR was found, based on binding within the linear range of the standard curve (between 20-80%). We then determined for the pooled pig saliva sample the amount of IR that occurred in the same fractions in which IR was also present for the OXT standard. The sum of IR in these co-occurring fractions was labeled ‘explained IR’, referring to IR that overlapped with the standard and therefore most probably stems from oxytocin. We then determined the amount of IR found in fractions of the pooled saliva sample where no IR was found in the OXT standard. The sum of IR in these fractions was labeled ‘unexplained IR’. We then calculated the proportion of unexplained IR (%) as: ((total IR – sum of IR in OXT Standard) / total IR ) * 100. The proportion of explained IR (%) was then calculated as: 100- proportion of unexplained IR. We found that 81.9% of the IR detected in the pooled pig saliva sample occurred in the same fraction in which IR was detected in the synthetic OXT sample (‘explained immunoreactivity’, see Supplementary Figure S2b). In addition, for both the pooled saliva sample and the pure OXT sample, the fraction in which the majority of IR was detected corresponds with the retention time of pure OXT in the HPLC chromatogram (see Supplementary Figure S2a) . The immunogram confirms that our method captures the majority of oxytocin and its metabolites and is suitable for measuring oxytocin in pig saliva.

# Supplementary Figures

**Supplementary Figure S1.** Parallelism for dilutions of pooled saliva samples (in orange) and kit standard curves (in blue) for oxytocin (A) and cortisol (B). The y-axis indicates sample optical density (b) / maximum binding optical density (b_0_). Regression lines indicate linear models predicting the expected sample concentrations as a function of binding.


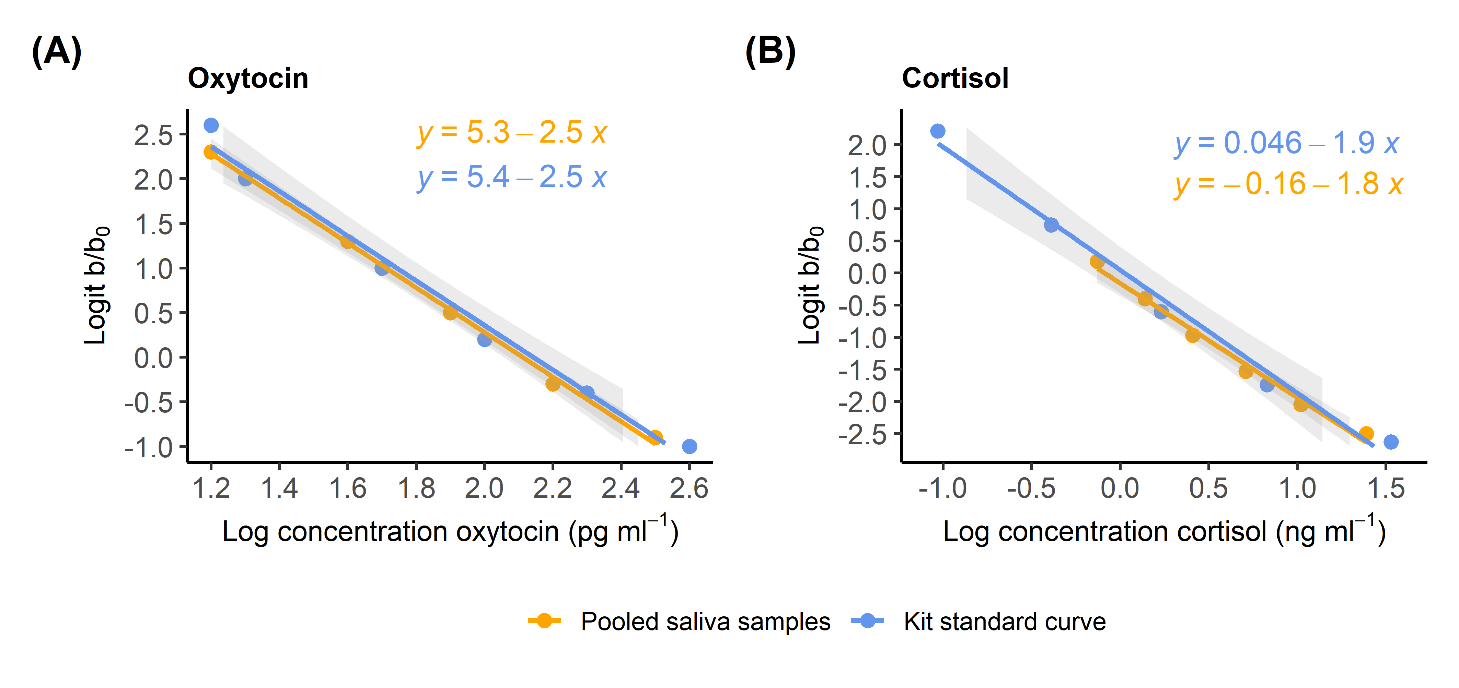


**Supplementary Figure S2.** Analytical validation of retention time of oxytocin in A) Pure oxytocin standard (Sigma-Aldrich, No. O6379) measured with HPLC in a chromatogram (UV extinction at 220 nm), and B) Extracted pig saliva samples and pure oxytocin standard from the EIA (Cayman chemicals, CAS Number 50-56-6), fractionated via HPLC and measured using the Cayman enzyme immuno-assay. Results indicate that the majority of immunoreactivity in extracted pig saliva samples occurs at the same retention times as the immunoreactivity in pure oxytocin standards and coincides with the retention time of pure OXT in the HPLC chromatogram.

**(A)**

**
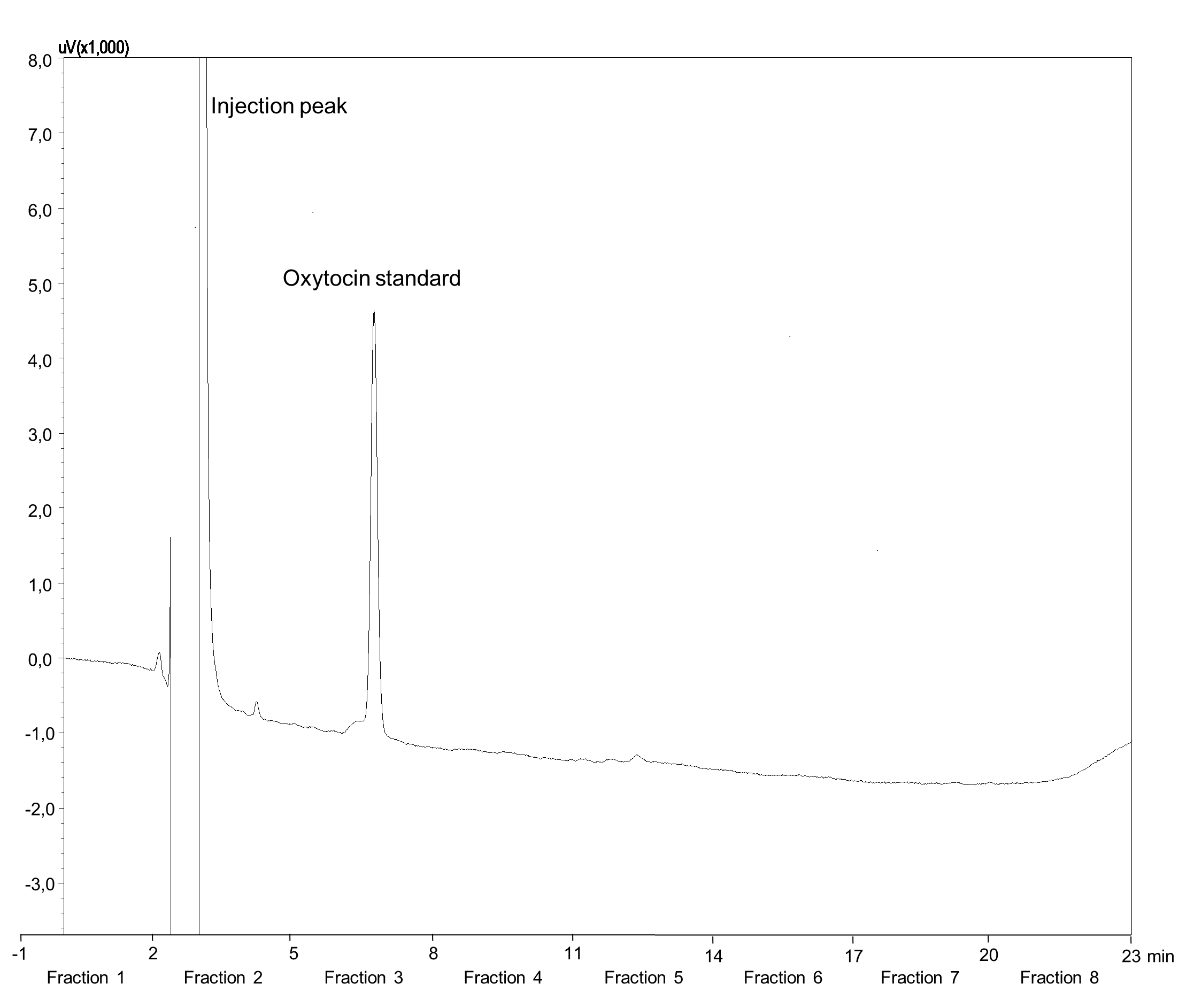
**

**(B)**

**
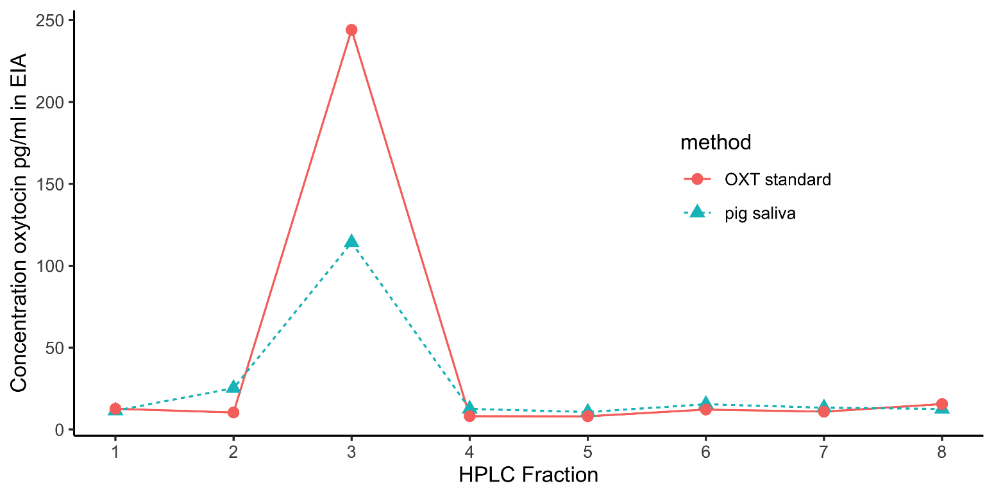
**

**Supplementary Figure S3.** Biological validation of salivary oxytocin concentrations in response to parturition. Salivary oxytocin was measured in n = 11 sows one day before and during parturition.


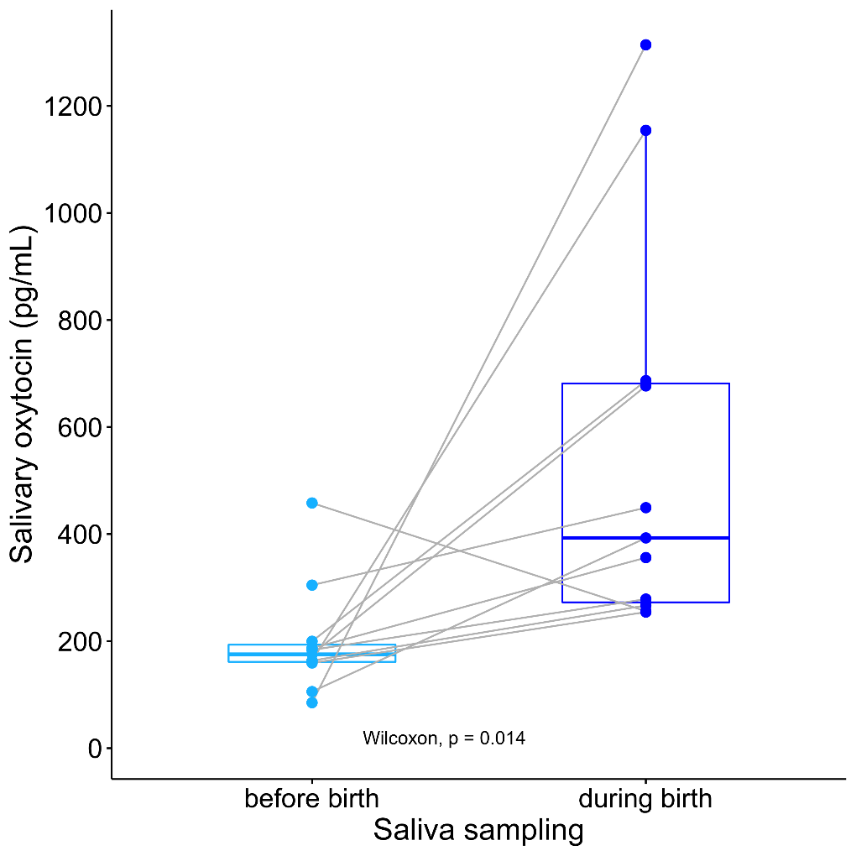


# Supplementary Video Files

Movie 1. Video clip showing group locomotor play during the play context, when pigs were given access to a hallway outside of their home pen for 30 minutes.

Movie 2. Video clip showing affiliative licking and nibbling behavior directed by several group members at pig 2, during her fifteen-minute reunion period after a brief social isolation.
